# Tunable Reflection through Size Polydispersity of Chiral-Nematic Liquid Crystal Polymer Particles

**DOI:** 10.3390/molecules28237779

**Published:** 2023-11-25

**Authors:** Tomoki Shigeyama, Kohsuke Matsumoto, Kyohei Hisano, Osamu Tsutsumi

**Affiliations:** Department of Applied Chemistry, Ritsumeikan University, 1-1-1 Nojihigashi, Kusatsu 525-8577, Japankmatsu@fc.ritsumei.ac.jp (K.M.)

**Keywords:** chiral-nematic liquid crystal, polymer particle, Bragg reflection, reflective coating, dispersion polymerization, circularly polarized light

## Abstract

Micro-sized chiral-nematic liquid crystal (N* LC) polymer particles have attracted considerable interest as versatile reflective colorants with selective circularly polarized light (CPL) properties. However, challenges in achieving the desired size distribution of N* LC particles have led to an incomplete understanding of their reflective characteristics. In this study, we successfully synthesized N* LC particles via dispersion polymerization, enabling precise control over size polydispersity by manipulating the composition of the polymerization solvent. Our investigation revealed that monodisperse N* LC particles displayed distinct reflection bands with high CPL selectivity, while polydisperse particles exhibited broader reflection with lower CPL selectivity. These findings underscore the potential to synthesize N* LC particles with tailored reflective properties using identical monomeric compounds. Furthermore, we demonstrated the production of multifunctional reflective colorants by blending N* LC particles with varying reflection colors. These discoveries hold significant promise for advancing the development of reflective colorants and anti-counterfeiting printing techniques utilizing micro-sized N* LC particles.

## 1. Introduction

Extensive research has focused on flexible polymer photonic materials with tunable optical functions, finding applications in optical sensors, displays, reflective coatings, and anti-counterfeiting printing [1,2,3,4]. Among these materials, chiral-nematic liquid crystals (N* LC) have received considerable attention. N* LC consists of nematic mesogens and chiral dopants, and in the N* LC phase, the mesogens align helically, resulting in a periodic distribution of the refractive index [5,6]. Consequently, N* LC materials exhibit tunable Bragg reflection with wavelength selectivity. The selective reflection wavelength (*λ*) can be estimated using the following equation:

*λ* = *n* · *P* sin θ.

Here, *n* represents the average refractive index of the material, *P* denotes the helical pitch of N* LC, and *θ* corresponds to the incidence angle. By controlling the concentration of chiral dopants, *P* can be adjusted, enabling the fabrication of N* LC materials with desired *λ*, namely, reflection colors. The helical alignment of mesogens in N* LC materials also leads to circularly polarized light (CPL) selectivity in their reflection. Exploiting these distinctive characteristics, reflective coatings and holographic materials have been developed by precisely manipulating the alignment of mesogens in N* LC materials [7,8,9].

In recent years, significant progress has been made in fabricating droplets or particles of N* LCs with controlled mesogenic alignment, unveiling their advanced optical functions [7,10,11,12,13,14,15,16,17,18,19,20,21,22]. The intriguing functionalities drive the development of various sizes of N* LC particles from sub-micrometer to millimeter scales via the utilization of various particle formation methods. These droplets or particles with a size of above 10 µm are typically prepared by suspending N* LCs in water via vortexing or microfluidic techniques, enabling precise control of their mesogenic alignment through surface interactions [23,24]. When the mesogens align parallel to the interface, a radial helical axis alignment is formed, leading to angular-independent Bragg reflection (retro-reflection) [10,22]. This unique optical property has been investigated in detail for low molecular weight N* LC droplets with a size of more than several 10 µm [10,11,12,13,14,15,16,17,18,19,20]. By contrast, the smaller micro-sized N* LC droplets or particles have gained great attention due to exhibiting practical applicability, including dispersibility in various materials, and the potential for large-area coating towards anti-counterfeiting inks and micro-sensors. A promising approach for synthesizing such smaller particles is the utilization of a polymerization technique, including suspension polymerization, dispersion polymerization, etc. Polymerized N* LC particles, in particular, offer enhanced thermal stability, ensuring the integrity of their shape and mesogenic alignment. Belmonte et al. successfully synthesized polydisperse N* LC polymer particles through suspension polymerization and demonstrated their effectiveness as reflective coatings and optical sensors [7,21,25,26,27]. Nevertheless, our current understanding of several-micrometer-sized N* LC particles and their reflective functions remains limited, with only a few studies conducted in this area. In particular, the impact of size dispersity on their properties has yet to be explored.

One of the characteristic reflective functions of N* LC particles is the photonic cross-communication phenomenon, which is inter-particle reflection [7,28]. The reflected light from the N* LC particle is further reflected by neighboring particles. Thus, the intensity of photonic cross-communication should be affected by the size polydispersity of particles. In this study, our focus was to investigate the impact of particle size polydispersity on the reflective functions of micro-sized N* LC particles. We recently reported the first successful synthesis of monodisperse N* LC particles via dispersion polymerization [29]. In this technique, polymerization takes place in a solution containing monomers, a dispersion stabilizer, and an initiator within a poor solvent for the produced polymer. As polymerization progresses, the polymer precipitates, resulting in the formation of micro-sized particles [30,31,32]. By manipulating the affinity of the polymerization solvent to the produced polymer, both particle size and polydispersity can be regulated [31]. In this work, we successfully produced micro-sized N* LC particles with different degrees of size dispersity through dispersion polymerization. These N* LC particles exhibited varying polydispersity and corresponding reflection functions. Our findings revealed that the degree of polydispersity had a significant influence on the sharpness of the reflection bands and the CPL selectivity. This knowledge is of utmost importance for the practical utilization of micro-sized N* LC particles as coating materials, paving the way for their application in diverse fields.

## 2. Results and Discussion

### 2.1. Synthesis and Characterization of N* LC Polymer Particles

**Figure 1:**
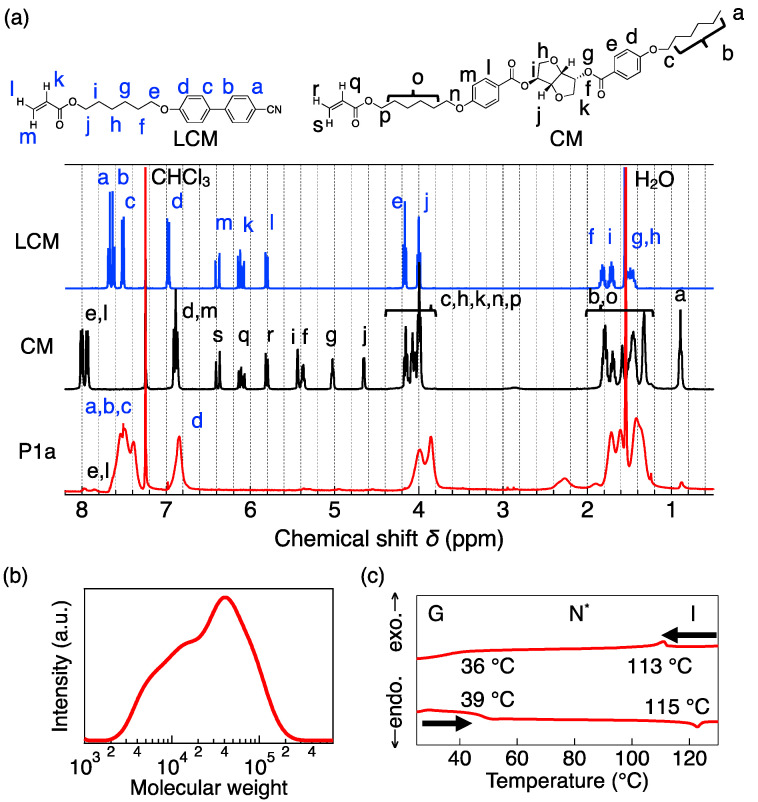
(**a**) ^1^H NMR spectra for monomers and polymer P1a in CDCl_3_ as a representative example: (blue) LCM, (black) CM, and (red) P1a. (**b**) Size-exclusion chromatogram of P1a. Molecular weight was calibrated with standard polystyrene. (**c**) DSC thermograms of P1a (2nd scan cycle). Scanning rate was 10 °C min^–1^. Abbreviations: G, glassy; N*, chiral nematic; I, isotropic.

**Figure 2:**
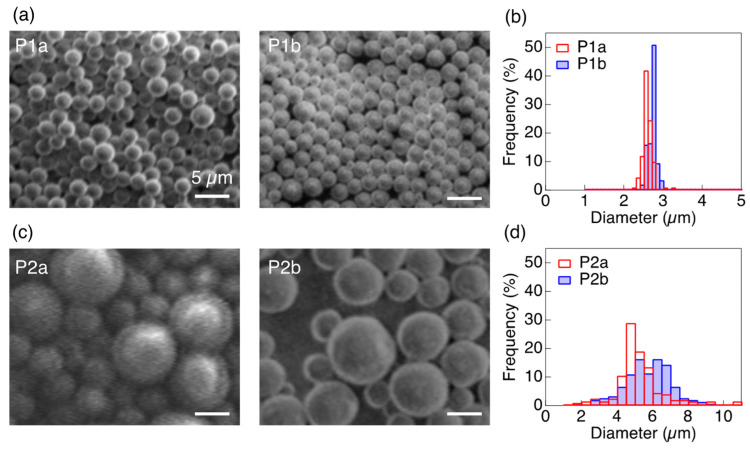
(**a**,**c**) SEM images and (**b**,**d**) histograms depicting the particle size distribution of N* LC polymer particles. Scale bars in the SEM images represent 5 µm.

N* LC particles were successfully synthesized via dispersion polymerization, with a conversion of ~50% (Table 1). We confirmed that the desired copolymer was obtained by dispersion polymerization (Figure 1a) and the copolymer composition was the same as the molar ratio of the polymerization mixture. The molecular weight and molecular weight distribution of the copolymers were determined by the SEC (Figure 1b) and are summarized in Table 1. In DSC measurements, during the cooling process, we observed a glass transition at 36 °C, accompanied by a phase transition peak at 113 °C, with an enthalpy change (Δ*H*) of 0.86 kJ/mol (Figure 2c). This Δ*H* aligns closely with the typical value reported for the I–N* phase transition of polymer LCs [33]. Identical thermal behavior was also observed in the heating process of the DSC thermogram. POM observations further support that the phase transition at 113 °C corresponds to the I–N* transition. Thus, we concluded that the copolymers synthesized in this study exhibit only the enantiotropic N* LC phase between 36 and 113 °C. SEM observations of the resulting polymer particles revealed the formation of spherical micro-sized particles under all polymerization conditions (Figure 2). The particle size and *CV* were determined by analyzing the SEM images and are summarized in Table 2. Consistent with a previous report [31], both the particle size and *CV* increased with higher volume ratios of DMF. For instance, particles synthesized in a solution with 50 vol% DMF (P1a and P1b) exhibited sizes of 2.5 µm with very small *CV* values (*CV* = 0.04). Conversely, particles obtained in a solution with 57 vol% DMF (P2a and P2b) displayed sizes of 5.3 µm with relatively larger *CV* values (*CV* = 0.3). We expected that these differences were due to the formation of unstable nuclei with increasing solubility of the produced polymers. In the mixed solvent system of MeOH and DMF, MeOH acted as a poor solvent, while DMF served as a rich solvent for the produced polymers. The affinity between the mixed solvent and the polymer intensified with the increasing DMF content, rendering the polymers formed at the initial dispersion polymerization stage unstable and prone to aggregation. This resulted in the formation of polydisperse nuclei, which subsequently grow larger during the growth process. Despite the constant amount of polymer produced in both P1 and P2 systems, irrespective of the solvent used, the particle size in the P2 series, characterized by a smaller number of nuclei, increased. Additionally, the polydispersity of the stable nuclei was maintained during the growth process, ultimately leading to the formation of large polydisperse microparticles in the P2 system. Notably, the particle size and *CV* remained independent of the CM ratio in the monomer mixture in all polymerization conditions.

### 2.2. Mesogenic Alignment in N* LC Polymer Particles

**Figure 3:**
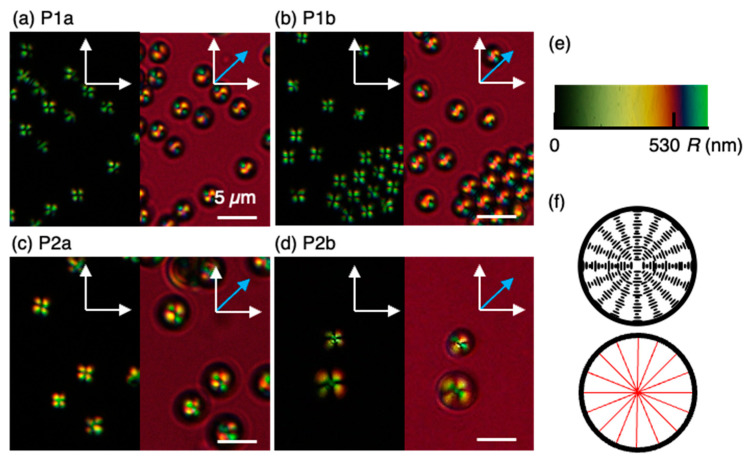
POM images of N* LC polymer particles dispersed in water observed without (**left**) and with the sensitive tint plate (**right**): (**a**) P1a, (**b**) P1b, (**c**) P2a, and (**d**) P2b. Scale bars represent 5 µm. White arrows show the direction of polarizers and blue arrows show the slow axis of the sensitive tint plate. (**e**) The interference color chart for POM observation. (**f**) Schematic illustrations of mesogenic alignment (**upper**) and helical axis alignment (**lower**) derived from POM results.

To explore mesogenic alignment, we performed polarized optical microscope (POM) observations of N* LC polymer particles suspended in water (Figure 3). In the aqueous medium, individual particles could be observed separately, preventing stacking and contacting with each other. This condition allowed for the observation of a clear optical texture for each particle. POM observation revealed that all particles exhibited a distinct texture characterized by a cross-shaped dark-field pattern known as the Maltese cross pattern, indicating a centrosymmetric mesogenic alignment [7,17]. The interference colors observed around the Maltese cross pattern were dark yellow (P1 series) and bright yellow (P2 series), suggesting that their retardations were around 100 nm and around 200 nm, respectively. The color difference might be due to the change in particle size. To further understand the alignment directions of the mesogens, we performed the POM observations using a sensitive tint plate with a 530 nm retardation and a one-dimensional slow axis. As depicted in Figure 3e, the interference color depends on their retardation; thus, we can identify the optical axis (here, the slow axis along the longer axis in the mesogen) due to the additive or subtractive effect resulting in higher or lower order interference colors when the slow axes of the mesogen and a tint plate are parallel or perpendicular, respectively. In the case of P1b, observed with the tint plate, a subtractive color (orange converted from dark yellow) was observed in the upper right and lower left areas, while an additive color (blue converted from dark yellow) was observed in the lower right and upper left areas. These results suggest that the mesogens in the polymer particles are aligned parallel to the interface, and the helical axis of the N* LC is radially aligned. The ideal mesogenic alignment speculated from these results is schematically depicted in Figure 3f. These POM observations are consistent with previous studies on the fabrication of low molecular weight N* LC droplets [7,17].

### 2.3. Reflection Properties of N* LC Polymer Particles

**Figure 4:**
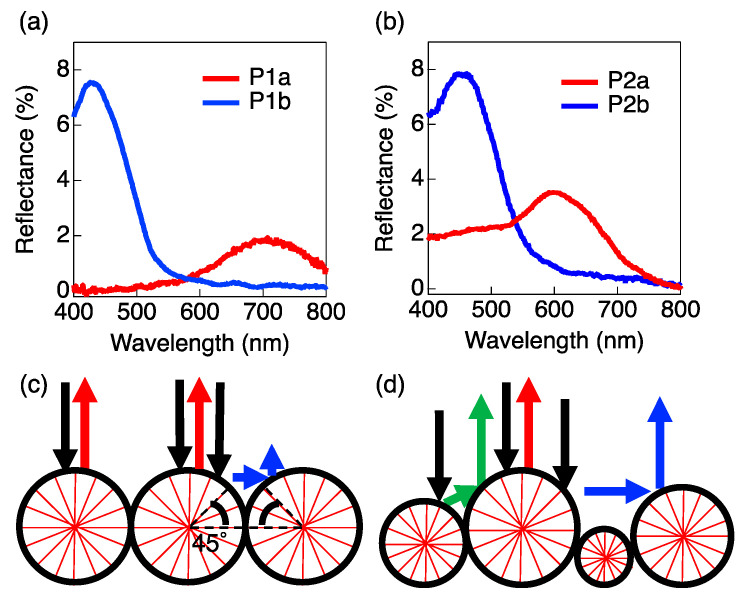
Reflection spectra of N* LC polymer particles: (**a**) P1 series and (**b**) P2 series. (**c**,**d**) Schematic illustration of the reflection mechanisms from N* LC polymer particles with radial helical axis alignment: (**c**) for monodisperse particles and (**d**) for polydisperse particles. The red lines in the particles indicate the direction of the helical axis within the particles. The black arrows represent the incident white light, the red arrows indicate retro-reflection light originating from the center of the particles, and the green and blue arrows represent photonic cross-communication.

The reflection spectra of N* LC polymer particles deposited onto carbon tape were measured using an integration sphere (Figure 4). All samples exhibited a distinct reflection band at a specific wavelength determined by the CM concentration. We examined the impact of size polydispersity on the selective reflection behavior. Particles P1a and P1b displayed well-defined and sharp reflection bands (Figure 4a). In contrast, particles P2a and P2b exhibited reflection peaks in a similar wavelength range as the P1 series, but their reflection bands appeared broader (Figure 4b). We attributed this broadening to photonic cross-communication [7,28]. We depict the schematic illustration of photonic cross-communication in Figure 4c,d. Due to the tilted alignment of the helical axis contributing to this phenomenon, the wavelength of photonic cross-communication is shorter than that of retro-reflection. In the case of monodispersed particles, uniaxial inter-particle reflections occur only when the helical axis is tilted by 45°, resulting in an exceedingly limited effective reflection area for the photonic cross-communication. Thus, the intensity of inter-particle reflections diminishes, making it undetectable in reflection spectra. Conversely, in polydisperse particle systems, the intensity of photonic cross-communication is higher due to the increased number of possible combinations of inter-particle reflections. Although P2b appears to exhibit sharp reflection, we believe that photonic cross-communication predominantly occurs in the ultraviolet region, out of the measurement range of our instrument.

### 2.4. Circular Polarization Selectivity of Photonic Cross-Communication

**Figure 5:**
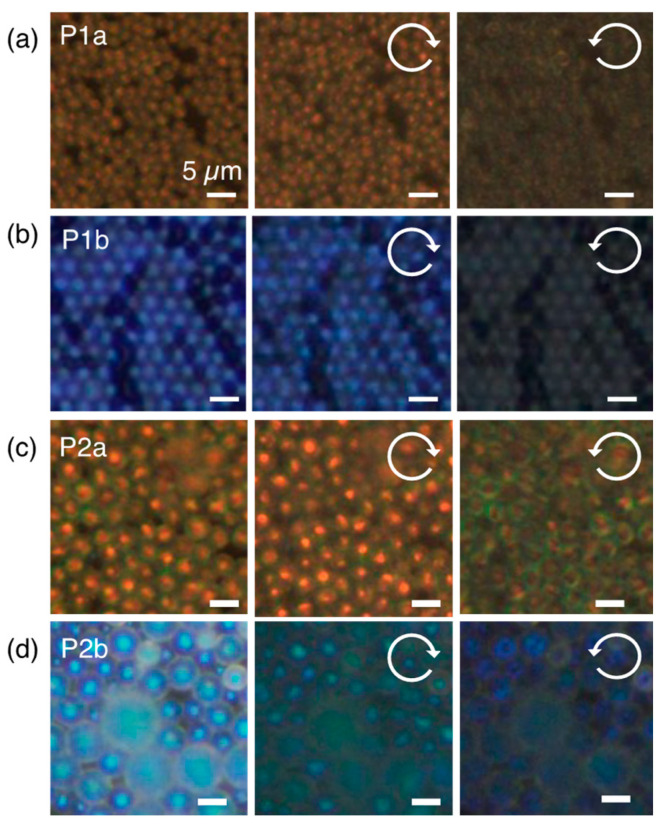
Reflection colors observed with epi-illuminated microscope: (**a**) P1a, (**b**) P1b, (**c**) P2a, and (**d**) P2b. Images depict the reflections without a filter (**left**), with RH-CPL transmission filter (**middle**), and with LH-CPL transmission filter (**right**). Scale bars indicate 5 µm.

To further investigate photonic cross-communication, we examined the reflections of N* LC polymer particles using epi-illuminated microscopy (Figure 5). Without a CPL filter, the reflection colors of each particle were consistent with the results obtained from the reflection spectroscopy. Both P1 and P2 series exhibited reflection colors corresponding to their respective helical pitches at the center of the particles. However, P2 also showed reflections of shorter wavelengths at the outer edge of the particles.

Next, we evaluated the CPL characteristics of the reflection light. Previous studies have shown that CM used in this study induces a right-handed (RH) helical structure and RH-CPL reflection [34]. Through observation of the reflected light through a RH-CPL transmission filter, we observed only the reflection color from the center for all particles, indicating that these reflections were retro-reflections from the particles. Interestingly, when using a left-handed (LH)-CPL transmission filter, these retro-reflections in the P1 series disappeared almost completely, while the P2 series still exhibited a green and deep blue reflection color at the outer edge, respectively. It has been previously demonstrated that CPL selectivity of N* LC is reduced under oblique incident light on the helical axis [35]. Via the above discussion regarding photonic cross-communication, which is derived from the tilted helical axis against the incident light, we concluded that the LH-CPL reflection in P2 particles contained reflection due to photonic cross-communication. Conversely, the absence of LH-CPL reflection in the P1 particles indicated no photonic cross-communication. These discussions are strongly supported by the results obtained from the reflection spectra.

Our findings indicate that reflection properties, such as reflection bandwidth and CPL selectivity, can be controlled by adjusting the polydispersity of N* LC particles. Monodisperse particles (P1 series) exhibit sharp reflection bands and high CPL selectivity, making them suitable for applications in reflective colorants, sensors, and holographic coatings. On the other hand, polydisperse particles (P2 series) display multiple reflection bands with varying CPL selectivity which can be utilized in advanced anti-counterfeiting printing.

### 2.5. Mixing N* LC Polymer Particles with Different Reflection Colors

**Figure 6:**
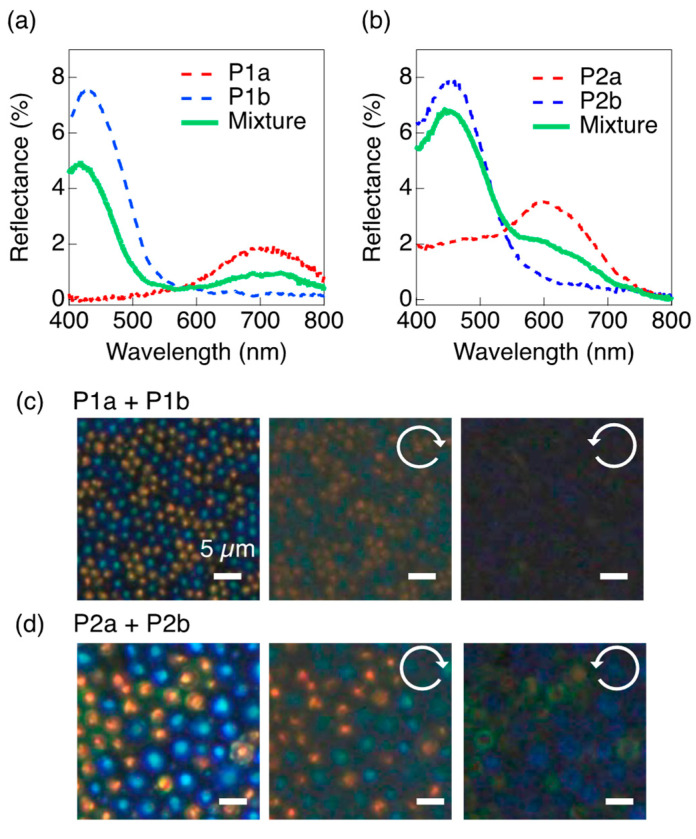
(**a**,**b**) Reflection spectra of N* LC polymer particles and their mixtures: (**a**) P1a (red), P1b (blue), and P1a + P1b (green); (**b**) P2a (red), P2b (blue), and P2a + P2b (green). (**c**,**d**) Epi-illuminated micrographs of the mixtures captured without a filter (**left**), with RH-CPL transmission filter (**middle**), and with LH-CPL transmission filter (**right**): (**c**) P1a + P1b and (**d**) P2a + P2b. Scale bars represent 5 µm.

To further demonstrate the capabilities of N* LC polymer particles in achieving advanced reflective functions, we conducted experiments to mix particles with different reflection colors. Aqueous dispersions of the particles were simply mixed in equal volumes. The mixture of P1a and P1b on carbon tape exhibited reflection spectra that were an addition of the spectra from each particle (Figure 6a). Microscopic examination under epi-illumination confirmed the homogeneous mixing of the particles, which independently reflected only the color corresponding to their helical pitches (Figure 6c). Importantly, this reflection originated solely from the center of the particles and exhibited pure RH-CPL. These results indicated that monodisperse N* LC particles did not display photonic cross-communication even when mixed with different reflection colors. Consequently, the reflection color of monodisperse particles can be easily modulated as a simple addition of colors.

On the other hand, the mixture of P2a and P2b demonstrated a more broadened reflection band (Figure 6b). Unlike the monodisperse particles, the spectral shape of this mixture did not simply result from the linear summation of individual particle spectra; instead, the reflection bands within the mixture exhibited asymmetrical broadening predominantly in the shorter wavelength region (400 to 550 nm). Epi-illumination microscopy revealed that this mixture exhibited the reflection colors at both the center and the edge of the particles (Figure 6d). Under CPL filters, the mixture reflected not only RH-CPL at the center but also LH-CPL at the edge. Specifically, a green reflection color was observed at the edge of P2a, while the edge of P2b exhibited a blue to deep blue reflection color. As mentioned, these observations were attributed to photonic cross-communication. Specifically, the occurrence of photonic cross-communication between P2a and P2b was accompanied by an increased intensity in a wider wavelength range compared to the individual cases of these particles. This suggests that P2b efficiently reflected light from P2a, thereby enhancing photonic cross-communication and increasing the reflectance in the shorter wavelength region.

These results clearly demonstrate that size dispersity can modulate the reflection color and CPL selectivity when mixing N* LC particles with different reflection colors. These findings open up new possibilities for creating advanced reflective coatings using N* LC particles. For instance, by combining LH- and RH-CPL reflective N* LC particles, holographic images can be achieved when viewed through circularly polarizing glasses.

## 3. Materials and Methods

### 3.1. Materials

**Scheme 1:**
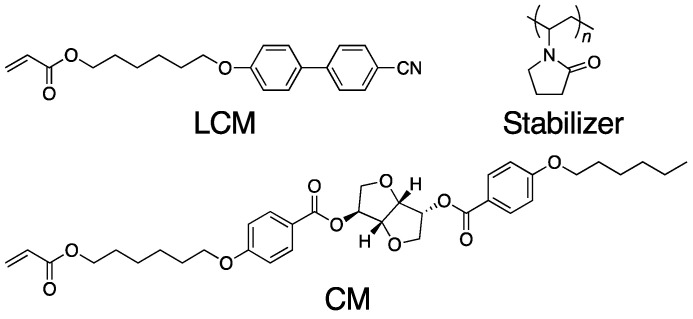
Molecular structures of liquid crystal monomer (LCM), chiral monomer (CM), and dispersion stabilizer (PVP) employed for synthesis of N* LC polymer particles.

The molecular structures of monomers and dispersion stabilizers are shown in Scheme 1. The base liquid crystal monomer (LCM) was kindly provided by Osaka Organic Chemical Industry Ltd.((Osaka, Japan)) and recrystallized from methanol (MeOH) prior to use. LCM usually shows a nematic liquid crystal phase in the polymer state [36]. The synthesis and purification of a chiral monomer (CM) have been previously reported [37]. The dispersion stabilizer, polyvinylpyrrolidone (PVP), and polymerization initiator, 2,2′-azobis(isobutyronitrile) (AIBN), were obtained from commercial suppliers and used as received. All solvents and other reagents used in this study were of reagent grade, commercially available, and used without further purification unless otherwise stated.

### 3.2. Preparation of N* LC Particles

LCM, CM, PVP, and AIBN were dissolved in a mixed solvent of *N*,*N*-dimethylformamide (DMF) and MeOH in a 30 mL Schlenk flask. The composition of the polymerization mixture is listed in Table 1. The solution was degassed by several freeze–pump–thaw cycles, backfilled with Ar gas, and stirred at 55 °C for 20 h. The resulting N* LC particles were isolated via filtration through a membrane filter (ADVANTEC T080A025A) with a pore size of 0.8 µm and washed with MeOH to remove the residual monomers, initiators, and excess amount of stabilizer to obtain the desired N* LC polymer particles in around 50% conversion.

### 3.3. Characterization of N* LC Particles

^1^H NMR spectra were recorded on a ECS-400 spectrometer (400 MHz, JEOL, Tokyo, Japan) in CDCl_3_. Chemical shifts were reported in parts per million (ppm), using the residual proton in the NMR solvent. Size-exclusion chromatography (SEC) was performed using LC-20AD (Shimadzu, Kyoto, Japan) equipped with a Shodex KF805 column and UV-vis detector (254 nm) at 40 °C, using THF as an eluent at a flow rate of 1 mL min^–1^. The molecular weights were calibrated using a polystyrene standard. The thermodynamic property was determined using differential scanning calorimetry (DSC, X-DSC7000, SII, Tokyo, Japan) at heating and cooling rates of 10 °C min^−1^. All analytical data confirmed that the desired polymers were obtained.

A suspension of N* LC polymer particles (1.0 mg mL^−1^, 0.2 mL) in water was deposited onto a pre-cleaned glass substrate (15 mm × 13 mm) and allowed to slowly evaporate at 5 °C overnight. The resulting polymer particles on the substrate were then transferred onto carbon tape for examination using a scanning electron microscope (SEM) (VE-8800, KEYENCE, Tokyo, Japan). SEM image analysis was conducted using image processing software (ImageJ 1.54f) to determine the average particle size and coefficient of variation (*CV*) [38]. A total of 200 particles were analyzed in the image analysis.

The reflection spectra were acquired using a UV-vis spectrometer (V-550, Jasco, Tokyo, Japan) equipped with an integration sphere (ISV-469, Jasco, Tokyo, Japan). A standard white plate (RS50, StellarNet, Tampa, FL, USA) was employed as a reference. Polarized optical microscopy and epi-illuminated microscopy were conducted using a BX51 microscope (Olympus, Tokyo, Japan) equipped with an epi-illumination source and a CPL filter (CP42HE, Edmund Optics Japan, Tokyo, Japan).

## 4. Conclusions

In this study, we successfully prepared micro-sized N* LC particles with controlled size dispersity through dispersion polymerization and systematically investigated their reflection properties. By precisely controlling the size dispersity using different polymerization solvents, we elucidated the significant influence of size dispersity on reflection characteristics. Our findings revealed that monodisperse particles exhibit distinct behavior devoid of photonic cross-communication, displaying sharp reflection bands with high CPL selectivity. In contrast, polydisperse particles exhibit pronounced photonic cross-communication, resulting in broader reflection bands with diverse CPL selectivity. Furthermore, we demonstrated the impact of polydispersity on reflection color and CPL selectivity by mixing N* LC particles of different colors. These discoveries not only enhance our understanding of N* LC particle systems but also hold significant implications for their practical applications in reflective colorants, anti-counterfeiting printings, micro-sensors, and other related fields. The development of our N* LC particles contributes to the advancement of tunable reflective colorant technologies.

## 5. Patents

The methodology for generating N* LC particles outlined in this paper has been filed as following patents by Ritsumeikan University: JP 2020-139135A (2020) and PCT/JP2022/019448 (2022).

## Figures and Tables

**Table 1:** Polymerization conditions for synthesis of N* LC particles.

Particle	LCM ^1,2^	CM ^1,2^	PVP ^1^	AIBN ^1^	Solvents (vol/vol)		Conversion (%)	*M*_n_ (*M*_w_/*M*_n_)
					DMF	MeOH		
P1a	97.6	2.4	130	4.0	50	50	53	15,000 (2.6)
P1b	96.2	3.8					51	15,000 (2.6)
P2a	97.6	2.4			57	43	45	13,000 (2.1)
P2b	96.2	3.8					49	13,000 (2.1)

^1^ Molar ratio of the materials in the polymerization mixture. ^2^ Total concentration of monomers in the polymerization solution was maintained at 7.3 wt%.

**Table 2:** Size, coefficient of variation, and selective reflection wavelength of N* LC polymer particles.

Particle	Diameter (µm)	*CV*	*λ* (nm)
P1a	2.5	0.04	700
P1b	2.6	0.04	420
P2a	5.3	0.3	590
P2b	5.3	0.3	440

Abbreviations: *CV*, coefficient of variation; *λ*, maximum wavelength of reflection.

## Data Availability

The data presented in this study are available in this article.
